# Absolute Neutrophil Count and Mean Platelet Volume in the Blood as Biomarkers to Detect Lung Cancer

**DOI:** 10.1155/2020/1371964

**Published:** 2020-04-22

**Authors:** Xuming Zhu, Yan Chen, Yubao Cui

**Affiliations:** Department of Clinical Laboratory, Wuxi People's Hospital Affiliated with Nanjing Medical University, Wuxi, Jiangsu Province, China

## Abstract

**Objective:**

Inflammation plays an extremely considerable role in the development and progression of malignancies. Absolute neutrophil count (ANC) and mean platelet volume (MPV) in blood are associated with various inflammatory conditions and resulted in independent prognostic factors for lung cancer. However, whether ANC and MPV can be diagnostic markers for lung cancer remains unknown. This retrospective study investigated the roles of ANC and MPV, either alone or combined, in diagnosing lung cancer.

**Methods:**

This study analyzed data from lung cancer patients and healthy individuals in Wuxi People's Hospital Affiliated with Nanjing Medical University. The Mann–Whitney *U*-test was performed to compare differences between lung cancer patients and healthy individuals. Spearman's correlation analysis was used to assess correlations. Receiver operating characteristic (ROC) curves were performed to determine diagnostic accuracy.

**Results:**

209 patients diagnosed with lung cancer and 236 healthy subjects were enrolled in this study. Levels of ANC and MPV increased in lung cancer patients compared with healthy individuals (*P* < 0.001). ANC had statistically significant negative weak correlation with albumin concentrations (*r* = ‐0.154, *P* = 0.026), and MPV had statistically significant negative weak correlation with total protein concentrations (*r* = ‐0.153, *P* = 0.027) in lung cancer patients. ANC and neutrophil-to-lymphocyte ratio had statistically significant positive correlation in both lung cancer patients (*r* = 0.756, *P* < 0.001) and healthy subjects (*r* = 0.639, *P* < 0.001). MPV and platelet-to-lymphocyte ratio had statistically significant negative weak correlation in both lung cancer patients (*r* = ‐0.242, *P* < 0.001) and healthy subjects (*r* = ‐0.325, *P* < 0.001). ANC had sensitivity (SEN) and specificity (SPE) of 0.512 and 0.809, respectively, and the area under the curve (AUC) with 95% confidence interval (95% CI) was 0.656 (0.603-0.710). SEN and SPE of MPV were 0.928 and 0.708, respectively, and the AUC (95% CI) was 0.913 (0.889-0.938). When ANC and MPV were combined, SEN and SPE became 0.842 and 0.835, respectively, and the AUC (95% CI) became 0.919 (0.895-0.943).

**Conclusions:**

Compared with ANC or MPV alone, the combination of ANC and MPV can improve diagnostic ability to distinguish lung cancer patients from healthy subjects.

## 1. Introduction

Lung cancer is one of the deadliest malignancies [1]. It accounts for 25% of cancer-related deaths worldwide, which is much higher than other cancers such as breast cancer, prostatic cancer, and colorectal cancer [2]. In 2019, the American Cancer Society estimates 116,440 and 111,710 new lung cancer cases with 24% and 23% of new deaths per year for men and women, respectively [3]. Neuron-specific enolase (NSE) is a commonly clinical marker of lung cancer. However, at many basic-level hospitals in China, it is hard for NSE to be performed due to relatively high expense. For these basic-level hospitals, it is necessary to find clinically accessible indicators such as blood routine parameters to replace NSE.

Inflammation plays an extremely considerable role in the development and progression of malignancies [4, 5]. Some inflammatory biomarkers can even play a role in the diagnosis of malignancies [6, 7]. For example, preoperative serum interleukin-6 in combination with conventional tests may be useful biomarkers to diagnose high-grade serous ovarian cancer [6]. The panel of interleukin-17A and chemokine CC ligand 20 exhibited a good performance in the diagnosis of early-stage colorectal cancer [7].

During the inflammatory reaction, neutrophils are stimulated and activated by inflammatory cytokines to promote their phagocytosis and bactericidal effects [8]. Some studies have proved that absolute neutrophil count (ANC), one of the blood routine parameters, can be an independent prognostic factor for advanced lung cancer [9, 10], but whether it can be a diagnostic marker for lung cancer remains unknown. The mean platelet volume (MPV), another blood routine parameter to be an indicator of platelet size and activity, has been proposed as a possible marker of platelet function and activation [11]. MPV is associated with various inflammatory conditions [12]. Like ANC, MPV can also be used as an independent prognostic factor for lung cancer [13]. However, whether MPV can be a diagnostic marker for lung cancer remains unknown. The aim of the current study is to investigate the roles of ANC and MPV, either alone or combined, in diagnosing lung cancer.

## 2. Materials and Methods

### 2.1. Study Population

We retrospectively analyzed data from participants at Wuxi People's Hospital Affiliated with Nanjing Medical University between August 2019 and November 2019. Patients diagnosed with lung cancer according to medical records were regarded as the case group, and healthy subjects were regarded as the control group. The exclusion criteria for the case group included diabetes mellitus, acute inflammation, coronary artery disease, kidney disease, and other cancers. This study was approved by the ethics committee of Wuxi People's Hospital Affiliated with Nanjing Medical University. Patient consent was waived due to the retrospective nature of this study. The data was maintained with confidentiality, and this study was conducted in accordance with the Declaration of Helsinki.

### 2.2. Laboratory Assays

Venous blood (5 mL) was collected from each participant in the morning and placed in EDTA-K2 anticoagulation tubes and drying tubes. Whole blood samples in EDTA-K2 anticoagulation tubes were analyzed in a Sysmex XE-5000 Automatic Hematology Analyzer (Sysmex Corp., Kobe, Japan) to detect whole blood routine parameters, including absolute neutrophil count (ANC), total number of red blood cell (RBC), platelet (PLT) count, and mean platelet volume (MPV). Blood samples in drying tubes were centrifuged at 3000 × g for 3 min, and serum samples were analyzed within 2 hours by a Beckman AU5800 Automatic Analyzer (Beckman Coulter Inc., CA, USA) to detect biochemical markers including total protein (TP), albumin (ALB), alanine transaminase (ALT), and creatinine (CR). Values of other hematological markers including neutrophil-to-lymphocyte ratio (NLR) and platelet-to-lymphocyte ratio (PLR) were calculated.

### 2.3. Statistical Analysis

All statistical analyses were performed using SPSS version 20.0 (SPSS Inc., Chicago, USA). Continuous data were presented as the median (interquartile range) because none of the continuous data complied with normal distribution under the Kolmogorov-Smirnova test. The Mann–Whitney *U*-test and chi-squared test were, respectively, performed to compare statistical differences for continuous data and male/female ratio between the case group and the control group. Spearman's correlation analysis was used to assess correlations between ANC, MPV, and biochemical markers and correlations between ANC, MPV, and other hematological measures (NLR, PLR). Sensitivity (SEN), specificity (SPE), and area under the curve (AUC) with 95% confidence interval (95% CI) in receiver operating characteristic (ROC) curves were performed to determine diagnostic accuracy. Values of *P* < 0.05 were considered statistically significant.

## 3. Results

### 3.1. Differences between the Case Group and the Control Group

**Figure 1:**
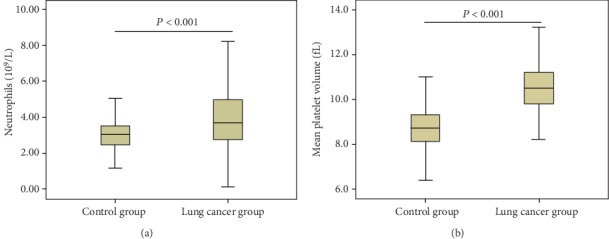
The levels of ANC and MPV in the lung cancer group and the control group: (a) ANC and (b) MPV.

We enrolled a total of 445 participants, including 209 lung cancer patients and 236 healthy subjects. As shown in Table 1, no statistically significant differences were present between the case group and the healthy subject group for age (*P* = 0.496) and gender (*P* = 0.232). Levels of biochemical markers such as TP and ALB were lower in the case group compared to the control group (*P* < 0.05). No statistically significant differences between groups were detected in ALT (*P* = 0.314) and CR (*P* = 0.196) levels. Levels of whole blood cell parameters such as ANC and MPV (Figure 1(a), Figure 1(b)) were higher in the case group compared to the control group (*P* < 0.001), while RBC count was lower in the case group (*P* < 0.001). No statistically significant differences between groups were detected in PLT count (*P* = 0.614).

### 3.2. Correlation Analysis

Results of correlation analysis between ANC, MPV, and biochemical markers are shown in Table 2. For ANC, it only had statistically significantly negative weak correlation with ALB in the case group (*r* = ‐0.154, *P* = 0.026) while it had no statistically significant correlation with all biochemical markers in the control group. For MPV, it only had statistically significant negative weak correlation with TP in the case group (*r* = ‐0.153, *P* = 0.027) while it had no statistically significant correlation with all biochemical markers in the control group.

Results of correlation analysis between ANC, MPV, and PLR are shown in Table 2. ANC and NLR had statistically significant positive correlation in both the case group (*r* = 0.756, *P* < 0.001) and the control group (*r* = 0.639, *P* < 0.001). MPV and PLR had statistically significant negative weak correlation in both the case group (*r* = ‐0.242, *P* < 0.001) and the control group (*r* = ‐0.325, *P* < 0.001).

### 3.3. Diagnostic Accuracy of ANC and MPV

**Figure 2:**
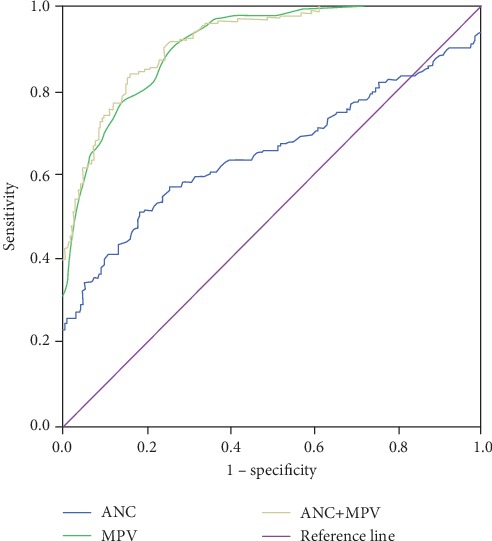
ROC curve analysis of the diagnostic performance of ANC and MPV, alone or combined.

The diagnostic accuracy of ANC and MPV to distinguish lung cancer from healthy subjects is presented in Figure 2 and Table 3. When the optimal cutoff point was 3.66 (×10^9^/L), ANC had SEN and SPE of 0.512 and 0.809, respectively, and the AUC (95% CI) was 0.656 (0.603-0.710). With an optimal cutoff point of 9.25 (fL), SEN and SPE of MPV were 0.928 and 0.708, respectively, and the AUC (95% CI) was 0.913 (0.889-0.938). Furthermore, when ANC and MPV were combined, SEN and SPE became 0.842 and 0.835, respectively, and the AUC (95% CI) increased to 0.919 (0.895-0.943).

## 4. Discussion

Cancer has been shown to mediate the immune response, which in turn plays an important role in tumor development, invasion, and metastasis [14]. Neutrophils are the most numerous innate immune cell type and represent 50-70% of the circulating leukocytes [15]. Neutrophils play an important role in the defense of the body, as well as in resolution and healing of inflammation [16]. Accumulating evidence also suggests the important role of neutrophils in cancer progression through their interaction with cancer and immune cells in blood and in the tumor microenvironment [17]. The current study showed that ANC was higher in lung cancer patients compared to healthy subjects, confirming the role of neutrophils in the progression of lung cancer.

MPV is also a useful indicator in some inflammatory diseases, and it has been associated with disease activity and severity of inflammation [18]. Released inflammatory mediators in cancer increase platelet activation, leading to a subsequent change in MPV [19]. The current study showed that MPV increased in lung cancer patients compared to healthy subjects, showing that the process of lung cancer may be associated with platelet activity and result in the variation of MPV.

ALB and TP are markers reflecting nutritional status [20]. Correlation analysis in the current study demonstrated that in lung cancer patients ANC and MPV were negatively weakly correlated with ALB and TP, respectively, which showed that the inflammatory stage of lung cancer might be related to nutritional insufficiency. NLR and PLR are important inflammatory indexes to reflect prognosis in several tumors [21]. The current study showed ANC and MPV had statistically significant correlations with NLR and PLR in both lung cancer patients and healthy subjects, confirming the inflammatory role of ANC and MPV in lung cancer.

This study showed that ANC and MPV alone had a diagnostic value to distinguish lung cancer from healthy subjects. Furthermore, the combined form of ANC and MPV can increase the diagnostic value compared to NEC and MPV alone, presenting its capability to be a clinically accessible indicator.

We acknowledge the fact that the current paper has some limitations. First, we did not divide lung cancer into small-cell lung cancer and non-small-cell lung cancer, ignoring their different pathogenic mechanisms. Second, we did not evaluate therapy measures involving surgery, chemotherapy, and radiotherapy, which could influence the levels of ANC and MPV in the blood.

Overall, compared with ANC or MPV alone, the combination of NEC and MPV can improve diagnostic ability to distinguish lung cancer patients from healthy subjects.

## Figures and Tables

**Table 1:** Characteristics of the study participants.

	Lung cancer group (*n* = 209)	Control group (*n* = 236)	*P* value
Male/female (*n*)	120/89	143/93	0.496
Age (years)	65 (58-70)	60 (48-75)	0.232
TP (g/L)	70.3 (64.8-74.1)	74.4 (72.2-76.7)	< 0.001
ALB (g/L)	40.3 (36.8-43.5)	44.3 (42.3-46.2)	< 0.001
ALT (U/L)	19 (13-29.5)	19 (15-28)	0.314
CR (*μ*mol/L)	72 (61-83)	71 (59-81)	0.196
ANC (×10^9^/L)	3.69 (2.71-4.98)	3.04 (2.46-3.52)	< 0.001
RBC (×10^12^/L)	4.17 (3.74-4.57)	4.75 (4.47-5.07)	< 0.001
PLT (×10^9^/L)	209 (170-259)	207 (177-240)	0.614
MPV (fL)	10.5 (9.8-11.2)	8.7 (8.1-9.4)	< 0.001

TP: total protein; ALB: albumin; ALT: alanine transaminase; CR: creatinine; ANC: absolute neutrophil count; RBC: red blood cell; PLT: platelet; MPV: mean platelet volume.

**Table 2:** Results of correlation analysis.

	Lung cancer group (*n* = 209)		Control group (*n* = 236)	
	*r*	*P* value	*r*	*P* value
ANC and TP	-0.06	0.385	0.022	0.736
ANC and ALB	-0.154	*0.026*	0.022	0.74
ANC and ALT	0.047	0.497	0.087	0.184
ANC and CR	0.026	0.713	0.054	0.407
ANC and NLR	0.756	<0.001	0.639	<0.001
ANC and PLR	0.190	0.006	0.051	0.438
MPV and TP	-0.153	0.027	0.086	0.187
MPV and ALB	-0.044	0.531	-0.017	0.790
MPV and ALT	0.039	0.577	-0.030	0.645
MPV and CR	0.074	0.286	-0.029	0.654
MPV and NLR	0.070	0.315	-0.100	0.126
MPV and PLR	-0.242	<0.001	-0.325	<0.001

NLR, neutrophil to lymphocyte ratio; PLR, platelet to lymphocyte ratio.

**Table 3:** Evaluation of diagnostic value.

Variables	Cut off point	Sensitivity	Specificity	AUC (95% CI)
ANC	3.66 (×10^9^/L)	0.512	0.809	0.656 (0.603-0.710)
MPV	9.25 (fL)	0.928	0.708	0.913 (0.889-0.938)
ANC + MPV		0.842	0.835	0.919 (0.895-0.943)

## Data Availability

This is a restropective paper. The data used to support the findings of this study are available from the corresponding author upon request.
